# Decreased serum apolipoprotein A1 level predicts poor prognosis of patients with de novo myelodysplastic syndromes

**DOI:** 10.1186/s12885-022-09248-2

**Published:** 2022-01-31

**Authors:** Cong Shi, Shengping Gong, An Wu, Shujun Yang, Duobing Zou, Yi Zhang, Ningning Wu, Chao Ma, Songqiu Shi, Ying Chen, Ying Wu, Xiaojiao Zheng, Zhenya Huang, Jianghua Ding, Guifang Ouyang, Qitian Mu

**Affiliations:** 1 Institute of Hematology, Ningbo First Hospital, No.59 Liuting Street, Ningbo, 315000 Zhejiang People’s Republic of China; 2 Cancer Radiotherapy and Chemotherapy Center, Ningbo First Hospital, No.59 Liuting Street, Ningbo, Zhejiang People’s Republic of China; 3 Department of Hematology, Ningbo First Hospital, No.59 Liuting Street, Ningbo, Zhejiang People’s Republic of China; 4 Department of Obstetrics and Gynaecology, Ningbo First Hospital, No.59 Liuting Street, Ningbo, Zhejiang People’s Republic of China; 5 Department of Hematology and Oncology, The Affiliated Hospital of Jiujiang University, No.57 Xunyang East Road, Jiujiang, 332000 Jiangxi People’s Republic of China

**Keywords:** Myelodysplastic syndromes, IPSS-R, Prognosis, Serum ApoA1, *TP53*

## Abstract

**Background:**

Myelodysplastic syndromes (MDS) is a group of heterogeneous myeloid clonal diseases originating from hematopoietic stem cells. It has been demonstrated that apolipoproteins A1(ApoA1) are associated with disease risk in many cancer types. However, there still lacks evidence regarding the link between ApoA1 and MDS. This study was designed to investigate the prognostic value of pretreatment ApoA1 levels in MDS patients.

**Methods:**

We retrospectively analyzed a cohort of 228 MDS patients to explore the prognostic value of the serum ApoA1 levels at diagnosis. Patients were divided into the high ApoA1 group and the low ApoA1 group. The prognostic significance was determined by univariate and multivariate Cox hazard models.

**Results:**

MDS patients with low ApoA1 levels had significantly shorter overall survival (OS, *P* < 0.0001) along with a higher frequency of *TP53* mutation (*P* = 0.002). Based on univariate analysis, age (≥ 60 years), gender (male), lower levels of hemoglobin (< 10 g/dl), HDL (≤0.91 mmol/L), higher bone marrow blast percentage (> 5%), higher IPSS-R scores and poorer karyotype were significantly associated with decreased OS. However, low ApoA1 level did not influence leukemia-free survival (LFS, *P* = 0.367). Multivariate Cox proportional hazards regression analysis indicated that low ApoA1 level (≤ 1.02 g/L) was also an independent adverse prognostic factor for OS in MDS (*P* = 0.034).

**Conclusions:**

Decreased ApoA1 level predicts a poor prognosis of MDS patients and thus provides a novel evaluation factor for them that is independent of the IPSS-R system.

## Background

Myelodysplastic syndromes (MDS), characterized by ineffective hematopoiesis that is manifested by morphologic dysplasia in hematopoietic cells and peripheral cytopenia(s), is a group of heterogeneous myeloid clonal diseases originating from hematopoietic stem cells with a high risk of transforming to secondary acute myeloid leukemia (AML) [1]. The prognosis of MDS is extremely heterogeneous, thus the Revised International Prognostic Scoring System (IPSS-R) was introduced to risk-stratify MDS patients in 2012 [2]. The scoring system mainly included the severity of hemocytopenia (anemia, thrombocytopenia, neutropenia, decreased hemoglobin content), increased bone marrow blasts, and cytogenetic factors. Recently, mutations such as *TP53*, *SRSF2*, *IDH2* and *ASXL1* were also demonstrated to be valuable in predicting the prognosis of MDS [3–5].

The tumor microenvironment interacts with tumor cells and plays a crucial role in tumorigenesis and development. By mediating complex signaling pathways, tumor microenvironment regulates the expression of various pro-inflammatory cytokines, chemokines, and angiogenic factors, all of which promote tumor growth, invasion and metastasis [6]. MDS also harbors a abnormal bone marrow microenvironment which contributes to the proliferation of tumor clones and eventually promotes the disease occurrence and development [7]. Evidence for the active lipid metabolism in tumor cells can be provided by quantifying the serum levels of lipid metabolites, such as apolipoprotein A1 (ApoA1), in cancer patients. Apo plays an important role in regulating lipid balance by transporting triglycerides, total cholesterol, and phospholipids, and is widely involved in the occurrence and development of tumors [8]. The role of apo in tumorigenesis and development may be achieved by promoting tumor invasion and metastasis, discounting anti-tumor drug delivery, and directly enhancing oxidative stress response [9–13]. In the past years, a correlation between serum ApoA1 level and disease risk has been observed in many cancer types. It has been also suggested that serum ApoA1 correlated with the survival rate of patients suffering from different types of tumors, such as gastric cancer, nasopharyngeal cancer, and colorectal cancer [14–16]. However, the prognostic value of serum ApoA1 for the overall survival of patients with MDS remains unclear. Therefore, we retrospectively analyzed the serum ApoA1 level at diagnosis to accurately delineate its meaningful prognostic value in MDS patients.

## Materials and methods

### Patients

Clinical and follow-up data of 228 patients who were newly diagnosed with MDS in Ningbo First Hospital from 2009 to 2019 were collected. Diagnosis and classification of MDS and leukemic transformation were determined according to the 2016 WHO classification [1]. Risk stratifications of MDS were made according to IPSS-R [2]. All laboratory examinations were investigated before treatment. Almost all patients received symptomatic and supportive treatment. Seventy-two patients acquired further treatments, among those 59 (25.9%) patients were treated with intensive chemotherapy, 18 patients (7.9%) with hemopoietic stem cell transplantation (HSCT) and 30 patients (13.2%) with hypomethylating agents. Patients with other types of malignant diseases were excluded. The patients had no concomitant disease that interacts with serum lipid levels (i. e. diabetes, hyperlipidemia, or metabolic syndrome) and hadn’t received hormone replacement therapy or use of any drugs known to affect lipid metabolism, such as HMG-CoA reductase inhibitors (e.g., simvastatin). Peripheral blood samples from 161 healthy donors were collected to serve as controls. Approval for the retrospective review of these records was obtained from the Ethics Committee of Ningbo First Hospital and was in accordance with the Declaration of Helsinki. Informed consent was obtained from all adult subjects or parents if subjects were under 18.

### Serum ApoA1 determination

Peripheral blood was drawn after strict fasting of at least 6 h. Serum ApoA1 level was measured using turbidimetric immunoassay. The reagents were tested by using Beckman’s ApoA1 kit withtheir instructions, under an automatic biochemical analyzer (Beckman AU5800).

### Morphology analysis

Morphology of MDS myeloid cells was observed through Wright-Giemsa stained bone marrow smears. It was evaluated subjectively by light microscopy at low power (10 × objectives) for overall quality and distribution, before further analysis at high power (100 × oil objectives) for the differential count.

### Cytogenetic analysis

BM cells were collected and cultured in RPMI-1640 medium supplemented with 20% newborn calf serum for 24 h. R-banded metaphases and the karyotypes were identified at least 20 metaphases for normal karyotype and at least 10 metaphases for abnormal karyotype according to the International System for Human Cytogenetic Nomenclature (2016) (ISCN2016) [17]. The karyotypes were grouped into five categories: very good, good, intermediate, poor and very poor according to the IPSS-R.

### Mutational analysis

Molecular analysis was performed as a part of the routine clinical work-up. Mutational analysis for 14 common genes of MDS including *NRAS*, *DNMT3A*, *SF3B1*, *IDH1*, *IDH2*, *TET2*, *EZH2*, *JAK2*, *CBL*, *ETV6*, *TP53*, *SRSF2*, *ASXL1* and *RUNX1* was performed using the next-generation sequencing. Variants with a variant allele frequency of <1% were excluded from the analysis. Multiplex PCR was used to amplify and construct sample library, high-throughput sequencing was performed on the Ion Proton platform, and bioinformatics analysis was performed with reference to PolyPhen, HG19, 1000 genomes, COSMIC, ClinVar, dbSNP databases. Gene mutation detection was completed by Kindstar Global Medical Laboratory (Wuhan, China).

### Statistical analysis

Statistical analyses were performed by SPSS 26.0. OS was calculated from the date of initial diagnosis of MDS to the date of death, last follow-up or acquiring allo-HSCT. Leukemia-free survival (LFS) was determined from the date of diagnosis to the date of leukemia transformation, last follow-up or acquiring allo-HSCT. OS and LFS were analyzed using the Kaplan-Meier method and were compared using the log-rank test. Multivariable analyses were performed using the Cox proportional hazard regression model. Differences in the distribution of continuous variables between categories were analyzed by Mann-Whitney *U* and categorical variables by Chi-squared test. The cutoff point of ApoA1 was calculated using the *X*-Tile software [18]. The optimal cutoff value for differences in survival was selected (the lowest *P*-value under the log-rank test) was 1.02 g/L. The *P*-value of < 0.05 was considered statistically significant.

## Results

### Patient characteristics

The data of 228 MDS patients, including 95 females and 133 males were collected over 10 yearswith a median age of 62 years (range 16–90 years). The median OS of these patients was 27 (range 0–125, 95% CI 15.952–38.048) months and 26 of them (11.4%) progressed to AML. Based on the 2016 WHO classification, all MDS patients were classified as follows: 23(10.1%) of MDS-SLD, 63(27.6%) of MDS-MLD, 15(6.6%) of MDS-RS, 59(25.9%) of MDS-EB1, 48(21.1%) of MDS-EB2, 6(2.6%) of MDS-del(5q) including del(5q) alone or with 1 additional abnormality except − 7 or del(7q), and 14 (6.1%) of MDS-U. Besides, 194 patients were stratified into IPSS-R risk groups as follows: 13 (6.7%) at very low risk, 36(18.6%) at low risk, 67(34.5%) at intermediate risk, 41(21.1%) at high risk and 37(19.1%) at very high risk. Of these, the median IPSS-R score was 4.5(1.0–10.0). Detailed information was provided in Table 1.

**Table 1:** Comparison of laboratory factors between MDS with low ApoA1 group and high ApoA1 group in 228 MDS patients

Variable	All patients	Low ApoA1 group (***n*** = 125)	High ApoA1 group (***n*** = 103)	statistics	***P value***
**Gender(n)**	228			χ2 = 10.638	0.001
Male/Female, n	133/95	85/40	48/55		
**Age [**years, median (quartile)**]**	62(51,73)	63(28 ~ 86)	61(16 ~ 90)	Z = -1.881	0.06
**BM Blast**[%, median (quartile)]	4(1,9)	6(0 ~ 19.5)	3(0 ~ 19)	Z = -2.718	0.007
**Peripheral Blood**					
NE [× 10^9^/L, median (quartile)]	1.2(0.7,2.1)	1.1(0 ~ 7.4)	1.3(1.1 ~ 6.9)	Z = -1.111	0.266
HB [g/L, median (quartile)]	7.5(6.2,9.9)	67(22 ~ 142)	88(50 ~ 142)	Z = -5.315	<0.0001
PLT [×10^9^/L, median (quartile)]	52(28,94)	46(4 ~ 332)	60(2 ~ 434)	Z = -2.809	0.005
ALB [g/L, median (quartile)]	39.4(35.4,43.0)	37.1(23.3 ~ 48.9)	42.0(18.3 ~ 60.2)	Z = -6.275	<0.0001
CHO [mmol/L, median (quartile)	3.56(2.78,4.26)	3.13(1.28 ~ 7.62)	4.07(2.01 ~ 8.92)	Z = -6.564	<0.0001
LDH [U/L, median (quartile)	205.5(167.0,269.8)	206(100 ~ 930)	203(94 ~ 618)	Z = -0.337	0.736
CRP [mg/L, median (quartile)	2.24(0.83,6.13)	2.7(0.33 ~ 33.69)	1.53(0.15 ~ 19.21)	Z = -2.477	0.013
HDL [mmol/L, median (quartile)	0.91(0.68,1.15)	0.73(0.09 ~ 1.48)	1.15(0.63 ~ 2.27)	Z = -10.316	<0.0001
LDL [mmol/L, median (quartile)	2.08(1.60,2.59)	1.84(0.32 ~ 5.07)	2.33(0.8 ~ 5.75)	Z = -5.151	<0.0001
ApoB [g/L, median (quartile)]	0.69(0.55,0.87)	0.63(0.23 ~ 1.29)	0.76(0.26 ~ 1.97)	Z = -4.670	<0.0001
ApoA1[g/L, median (quartile)]	1.00(0.82,1.18)	0.84(0.34 ~ 1.02)	1.19(1.03 ~ 2.36)	Z = -12.989	<0.0001
**2016 WHO classification**				χ2 = 14.801	0.039
MDS-SLD, % (n/n)	10.1% (23/228)	6.4% (8/125)	14.6% (15/103)		
MDS-MLD, % (n/n)	27.6% (63/228)	25.6% (32/125)	3.0% (31/103)		
MDS-RS-SLD, % (n/n)	2.2% (5/228)	1.6% (2/125)	2.9% (3/103)		
MDS-RS-MLD, % (n/n)	4.4% (10/228)	3.2% (4/125)	5.8% (6/103)		
MDS-5q-, % (n/n)	2.6% (6/228)	1.6% (2/125)	3.9% (4/103)		
MDS-EB1, % (n/n)	25.9% (59/228)	27.2% (34/125)	24.3% (25/103)		
MDS-EB2, % (n/n)	21.1% (48/228)	28.8% (36/125)	11.7% (12/103)		
MDS-U, % (n/n)	6.1% (14/228)	5.6% (7/125)	6.8% (7/103)		
**IPSS-R cytogenetic risk group**				χ2 = 2.738	0.603
Very good, % (n/n)	1.0% (2/194)	1.0% (1/97)	1.0% (1/97)		
Good, % (n/n)	63.9% (124/194)	59.8% (58/97)	68.0% (66/97)		
Intermediate, % (n/n)	20.6% (40/194)	24.7% (24/97)	16.5% (16/97)		
Poor, % (n/n)	5.2% (10/194)	4.1% (4/97)	6.2% (6/97)		
Very poor, % (n/n)	9.3% (18/194)	10.3% (10/97)	8.2% (8/97)		
**IPSS-R risk category**				χ2 = 10.349	0.035
Very low, % (n/n)	6.7% (13/194)	3.1% (3/97)	10.3% (10/97)		
Low, % (n/n)	18.6% (36/194)	14.4% (14/97)	22.7% (22/97)		
Intermediate, % (n/n)	34.5% (67/194)	34.0% (33/97)	35.1% (34/97)		
High, % (n/n)	21.1% (41/194)	22.7% (22/97)	19.6% (19/97)		
Very high, % (n/n)	19.1% (37/194)	25.8% (25/97)	12.4% (12/97)		
**IPSS-R score** [median (quartile)]	4.5(3.0,6.0)	4.5(2.0 ~ 10.0)	4.0(1.0 ~ 9.0)	Z = -3.188	0.001
**Gene mutation, % (n/n)**	64.1% (41/64)	71.4% (20/28)	58.3% (21/36)	χ2 = 1.173	0.279
**Leukemia transformation, % (n/n)**	11.4% (26/228)	12.8% (16/125)	9.7% (10/103)	χ2 = 0.534	0.465
**Complex karyotype, % (n/n)**	18.0% (35/194)	21.6% (21/97)	14.4% (14/97)	χ2 = 1.708	0.191
**With cardiovascular comorbidity, % (n/n)**	21.9% (50/228)	21.6% (27/125)	22.3% (23/103)	χ2 = 0.018	0.895

*Abbreviations*: *BM* bone marrow, *NE* neutrophil, *HB* hemoglobin, *PLT* platelet, *ALB* albumin, *CRP* C reactive protein, *CHO* cholesterol, *LDH* lactic dehydrogenase, *HDL* high-density lipoprotein, *ApoB* apolipoprotein B, *LDL* low-density lipoprotein, *ApoA1* apolipoprotein A1, *MDS-SLD* MDS with single lineage dysplasia, *MDS-MLD* MDS with multilineage dysplasia, *MDS-RS-SLD* MDS with ring sideroblasts and single lineage dysplasia, *MDS-RS-MLD* MDS with ring sideroblasts and multilineage dysplasia, *MDS-EB1* MDS with excess blasts 1, *MDS-EB2* MDS with excess blasts 2, *MDS-U* unclassifiable, *IPSS-R* Revised International Prognostic Scoring System

### The relationship between ApoA1 level and other factors in clinics and laboratory

**Fig. 1:**
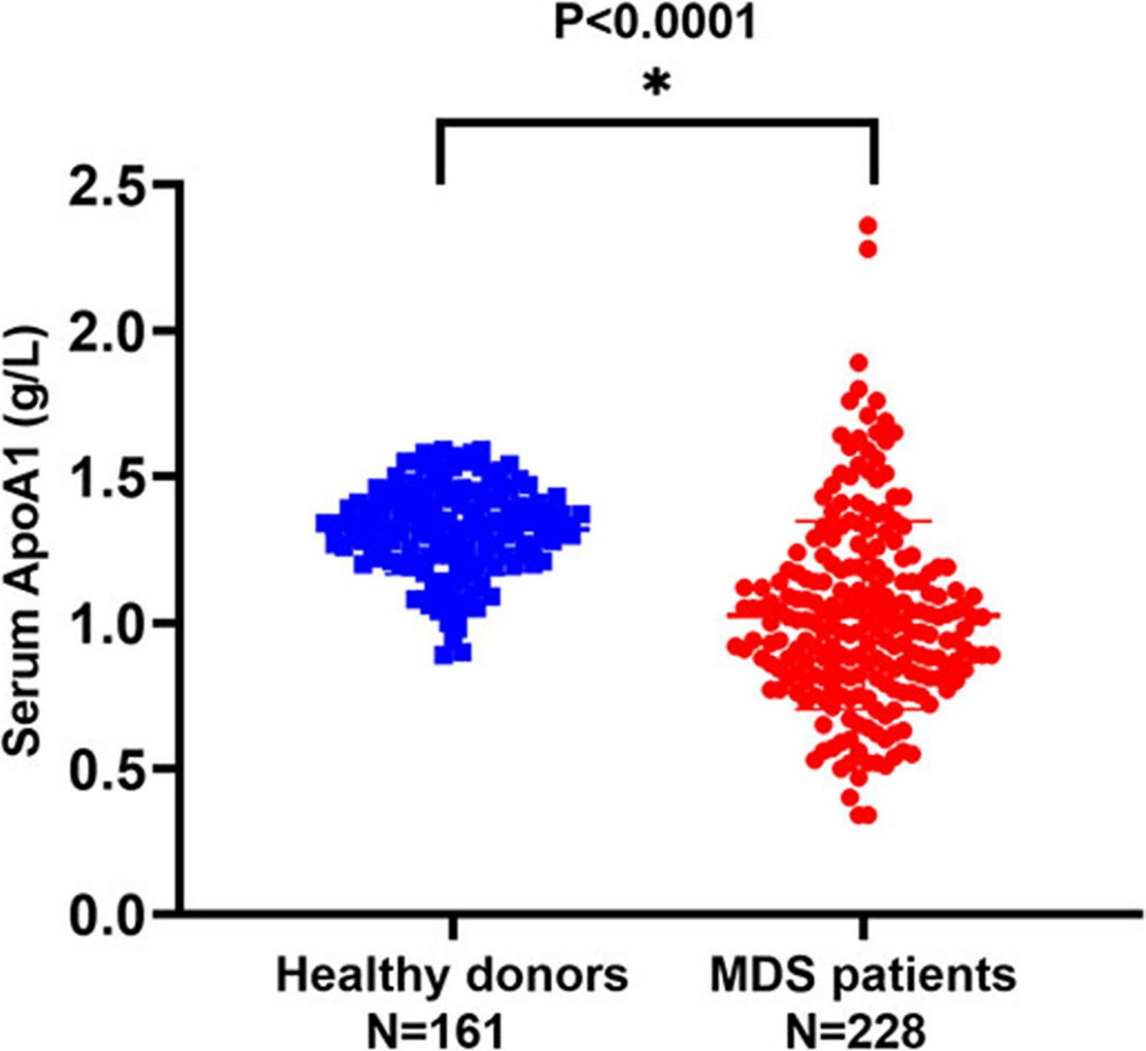
Comparison of serum ApoA1 between 161 healthy donors and 228 MDS patients

In our cohort, the median ApoA1 level in 228 MDS patients was lower than that in 161 healthy donors (1.00 g/L vs. 1.33 g/L, *P* < 0.0001; Fig. 1). Furthermore, MDS patients were divided into two groups to analyze the correlation between ApoA1 level and other clinical and laboratory characteristics. It showed that, compared with the high ApoA1 group, the low ApoA1 group had significantly more counts of BM blast (*P* = 0.007), higher levels of CRP (*P* = 0.013) and fewer counts of HB (*P* < 0.0001), PLT (*P* = 0.005), ALB (*P* < 0.0001), CHO (*P* < 0.0001), HDL (*P* < 0.0001), LDL (*P* < 0.0001), and ApoB (*P* < 0.0001) along with higher risk distribution in terms of IPSS-R (*P* = 0.035). Additionally, the WHO subtype between these two groups had a significant difference (*P* = 0.039). There were no significant differences in other factors between the two groups (Table 1).

### Low ApoA1 level was accompanied with a higher frequency of *TP53* mutation

**Fig. 2:**
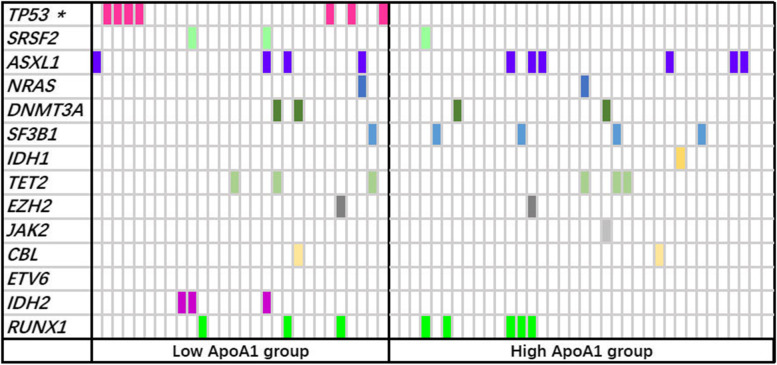
Mutation spectrum of 14 common genes in 64 MDS patients. Each column represents an individual patient sample, and each coloured cell represents a mutation of the gene. There was a significant difference between the two groups in *TP53* gene mutation (*P* = 0.002)

The mutation profile of 14 dominant genes were detected in 64 patients, 41(64.1%) of whom harbored at least one mutated gene. The mutation rates of the 14-target genes as follows: *ASXL1* (15.6%), *TP53* (10.9%), *RUNX1* (12.5%), *SF3B1* (7.8%), *TET2* (7.8%), *DNMT3A*(6.3%), *IDH2* (4.7%), *SRSF2* (4.7%), *NRAS*(3.1%), *EZH2* (3.1%), *CBL* (3.1%), *IDH1* (1.6%), *JAK2* (1.6%) and *ETV6* (0.0%) (Fig. 2). On the whole, the ApoA1 deficient group harbored a higher mutation rate in comparison with the ApoA1 proficient group, albeit the difference was not statistically significant (71.4% vs. 58.3%, *P* = 0.279). Of note, the low ApoA1 group showed a higher mutation frequency of *TP53* compared with the high ApoA1 group (25.0% vs. 0.0%, *P* = 0.002). There was no difference between the two groups in other 13 gene mutation (data not shown).

### Low ApoA1 level was associated with a poor prognosis

**Fig. 3:**
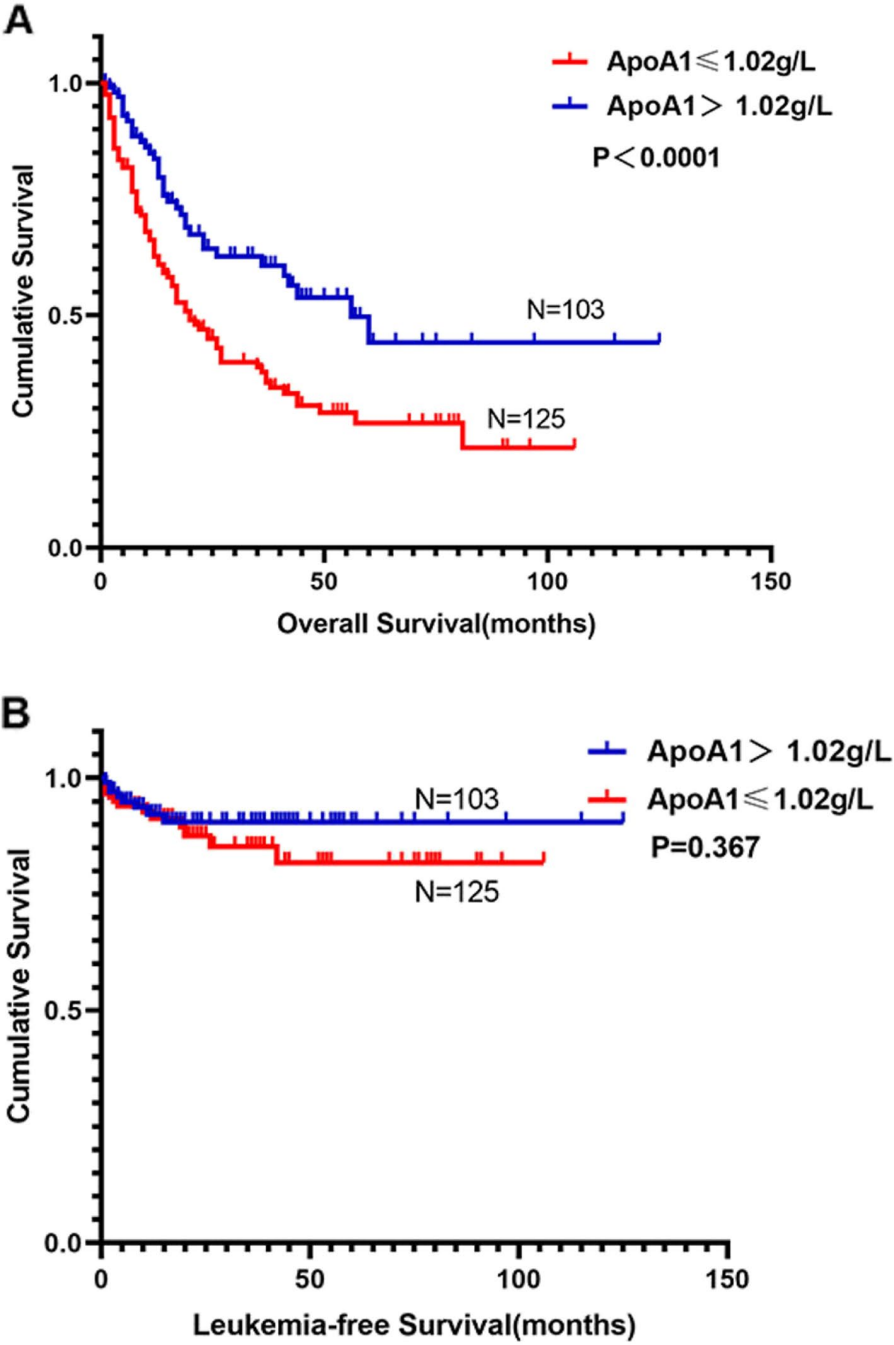
Overall survival and leukemia-free survival of MDS patients according to the stratified analysis of ApoA1. **A** Overall survival of 228 patients with primary MDS was stratified by ApoA1 ≤ 1.02 g/L vs. ApoA1 > 1.02 g/L (*P* < 0.0001). **B** Leukemia-free survival of 228 patients with primary MDS was stratified by ApoA1 ≤ 1.02 g/L vs. ApoA1 > 1.02 g/L (*P* = 0.367)

Compared with the high ApoA1 group, the median OS in the low ApoA1 group was significantly shorter (19 months vs 56 months, *P* < 0.0001; Fig. 3A). However, when it comes to the LFS, the difference between the two group was statistically insignificant (*P* = 0.367; Fig. 3B).

In univariate analysis, the OS was adversely associated with older age (≥60 years, *P* < 0.0001), male gender (*P* = 0.012), higher-risk IPSS-R cytogenetic (*P* = 0.012), higher BM blast percentage (> 5%, *P* < 0.0001), higher IPSS-R score (*P* < 0.0001), lower levels of HB(< 10 g/dl) (*P* = 0.005), lower levels of HDL (≤0.91 mmol/L) (*P* = 0.002),and ApoA1 (≤1.02 g/L, *P* < 0.0001).

Multivariate analyses showed that older age (≥60 years, *P* < 0.0001), higher BM blast percentage (> 5%, *P* < 0.0001), higher-risk IPSS-R cytogenetic (*P* = 0.005), were adverse factors while a low ApoA1 level was a significant prognostic factor for worse OS (*P* = 0.034) (Table 2). Therefore, decreased serum ApoA1 could predict a poor prognosis of MDS patients independent of the IPSS-R.

**Table 2:** Univariate and multivariate analyses of different prognostic parameters for overall survival of 228 patients with MDS

Variables	Univariate analysis for OS		Multivariate analysis for OS		
	***P-value***	95%CI	***P-value***	***HR***	95%CI
Age ≥ 60(years)	< 0.0001	12.429–21.571	< 0.0001	2.679	1.697–4.232
Gender (male)	0.012	14.393–27.607	0.059	1.542	0.984–2.417
HB < 10 g/dl	0.005	15.478–28.522	0.101	0.647	0.384–1.089
NE < 0.8 × 10^9^/L	0.101	10.024–27.976	0.885	1.035	0.653–1.639
PLT < 100 × 10^9^/L	0.121	12.288–35.712	0.204	0.707	0.414–1.208
BM blast > 5%	< 0.0001	9.280–18.720	< 0.0001	3.160	2.033–4.912
IPSS-R cytogenetic risk group	0.012	23.159–48.841	0.005	1.346	1.091–1.660
IPSS-R score	< 0.0001	23.159–48.841	–	–	–
With cardiovascular comorbidity	0.710	12.434–61.566	–	–	–
HDL ≤ 0.91 mmol/L	0.002	12.286–25.714	0.584	1.175	0.659–2.093
ApoA1 ≤ 1.02 g/L	< 0.0001	12.486–25.514	0.034	1.847	1.047–3.258

*Abbreviations*: *HB* hemoglobin, *NE* neutrophil, *PLT* platelet, *BM* bone marrow, *IPSS-R* Revised International Prognostic Scoring System, *HDL* high-density lipoprotein, *ApoA1* apolipoprotein A1

## Discussion

Based on the obtained results, we showed that pretherapy serum ApoA1 at a low level was associated with higher BM blast percentage, higher IPSS-R scores, higher *TP53* mutation rate, higher CPR levels as well as lower levels of HB, PLT, ALB, CHO, HDL, LDL and ApoB. We observed that ApoA1 was closely correlated with HDL. We have added HDL to our cohort. Decreased serum ApoA1 levels correlated with a shorter survival period in MDS, indicating that lower serum ApoA1 level reflects a poor prognosis in MDS patients. The Cox regression analysis revealed that the ApoA1 level was an independent prognostic factor for MDS patients.

The metabolic patterns of tumor cells, including lipid metabolism are different from those of normal cells. It has been demonstrated that lipids play an important role in the occurrence and development of malignant tumors. ApoA1 is synthesized predominantly in the liver and the small intestine, which is the predominant protein of plasma HDL [19]. ApoA1 not only participates in fat metabolism by regulating the cholesterol level in cells, but also shows innate immune activity and participates in the occurrence and development of tumors. For instance, ApoA1 takes part in the immunomodulatory effects of tumor microenvironment by enhancing treg response [20]. In addition, decreased level of ApoA1 is associated with tumors and has great potential for the early diagnosis, prognosis and therapeutic application of tumors. In the mature immune system, ApoA1 is activated and involved in anti-tumor [21]. A reported study demonstrated that in the tumor microenvironment, ApoA1 worked as a potent immunomodulatory agent by transforming tumor-associated macrophages from a pro-tumor to an antitumor phenotype. In vivo experimental results showed that ApoA1 was transformed from pro-tumor M2 macrophages to anti-tumor M1 phenotypes, and tumors were infiltrated by cytotoxic cells [22]. Lower serum ApoA1 levels even had a practical function to predict the recurrence of breast cancer [23]. Research on nasopharyngeal carcinoma has shown that serum ApoA1 level higher than 1.025 g/L is an independent predictor of longer overall survival, less local recurrence or distant metastasis in patients [24]. In conclusion, ApoA1 affects tumor growth and its deficiency may favor tumor progression. Similarly, our study showed that ApoA1 less than 1.02 g/L is correlated with poor OS and serves as an independent prognostic factor for survival. However, the role of ApoA1 in carcinogenesis is not well understood. Further, it was demonstrated in our cohort that MDS patients with low ApoA1 harbored higher BM blast percentage, lower HB and PLT levels and especially higher IPSS-R score.

*TP53* gene, located in the 17p13 chromosomal region is one of the major tumor suppressor genes and is often inactivated by deletion and/or mutation in many tumors, including hematologic malignancies [25]. The mutation rate of *TP53* was 5–10% in MDS [26]. *TP53* mutation in MDS is strongly associated with poor treatment outcomes [27]. *TP53* is known to play a role in lipid metabolism [28]. Goldstein et al. [29] found that *TP53* had the role in enhancing lipid catabolism while inhibiting its anabolism. In our cohort, we found that ahigh *TP53* gene mutation rate was correlated with the decrease of serum ApoA1 in MDS. Due to applying a 14-mutations panel to do the MDS mutation screening, the detected gene mutation rate is low, which is in line with the study of Mengyi Du [30].

It is well known that IPSS-R was widely used in measuring the prognosis of MDS. Although ApoA1 was reported to be a prognostic factor in several malignancies, to the best of our knowledge, an association between ApoA1 and the prognosis of MDS patients has not been reported to date. In this study, we found that the ApoA1 level in MDS patients was lower than that in controls, and was proved to be an independent predictor of OS.

Furthermore, ApoA1 could function as an independent prognostic factor of MDS, also it is a common and convenient indicator in pretreatment examination. In addition, this study provides a new idea for the prognostic evaluation of MDS, and provides a potential therapeutic target.

## Conclusions

We demonstrated that decreased ApoA1 level was accompanied by a higher frequency of *TP53* mutation and was associated with a poor prognosis in MDS patients. ApoA1 as a prognostic factor could provide convenience for evaluating the prognosis of MDS patients and be a useful supplement to IPSS-R. Thus, lipid metabolism-oriented therapeutics might be promising strategies for MDS patients. As this study is a retrospective analysis, it is only valid for generating a hypothesis, and the value of ApoA1 should be validated in large prospective trials.

## Data Availability

The data that support the findings of this study are available from Ningbo First Hospital but restrictions apply to the availability of these data, which were used under license for the current study, and so are not publicly available. Data are however available from the authors upon reasonable request and with permission of Ningbo First Hospital. Cong Shi, the first author, should be contacted if someone wants to request the data from this study.

## Abbreviation

MDS: Myelodysplastic syndromes
ApoA1: Apolipoprotein A1
OS: Overall survival
LFS: Leukemia-free survival
IPSS-R: Revised International Prognostic Scoring System
AML: Acute myeloid leukemia
IPSS: International Prognostic Scoring System
WHO: World Health Organization
BM: Bone marrow
HSCT: Hemopoietic stem cell transplantation
ISCN2016: International System for Human Cytogenetic Nomenclature (2016)
NE: Neutrophil
HB: Hemoglobin
PLT: Platelet
ALB: Albumin
CHO: Cholesterol
CRP: C reactive protein
LDH: Lactic dehydrogenase
HDL: High-density lipoprotein
ApoB: Apolipoprotein B
LDH: Low-density lipoprotein
MDS-SLD: MDS with single lineage dysplasia
MDS-MLD: MDS with multilineage dysplasia
MDS-RS-SLD: MDS with ring sideroblasts and single lineage dysplasia
MDS-RS-MLD: MDS with ring sideroblasts and multilineage dysplasia
MDS-EB: MDS with excess blasts
MDS-U: MDS with unclassifiable

## References

[1] Arber DA, Orazi A, Hasserjian R (2016). The 2016 revision to the World Health Organization classification of myeloid neoplasms and acute leukemia. Blood.

[2] Greenberg PL, Tuechler H, Schanz J (2012). Revised international prognostic scoring system for myelodysplastic syndromes. Blood.

[3] Tefferi A, Lasho TL, Patnaik MM (2017). Targeted next-generation sequencing in myelodysplastic syndromes and prognostic interaction between mutations and IPSS-R. Am J Hematol.

[4] Arbab Jafari P, Ayatollahi H, Sadeghi R (2018). Prognostic significance of SRSF2 mutations in myelodysplastic syndromes and chronic myelomonocytic leukemia: a meta-analysis. Hematology (Amsterdam, Netherlands).

[5] Lin P, Luo Y, Zhu S (2018). Isocitrate dehydrogenase 2 mutations correlate with leukemic transformation and are predicted by 2-hydroxyglutarate in myelodysplastic syndromes. J Cancer Res Clin Oncol.

[6] Ji Z, He L, Regev A (2019). Inflammatory regulatory network mediated by the joint action of NF-kB, STAT3, and AP-1 factors is involved in many human cancers. Proc Natl Acad Sci U S A.

[7] Montes P, Bernal M, Campo LN (2019). Tumor genetic alterations and features of the immune microenvironment drive myelodysplastic syndrome escape and progression. Cancer Immunol Immunother.

[8] Melvin JC, Holmberg L, Rohrmann S (2013). Serum lipid profiles and cancer risk in the context of obesity: four meta-analyses. J Cancer Epidemiol.

[9] Dos Santos CR, Domingues G, Matias I (2014). LDL-cholesterol signaling induces breast cancer proliferation and invasion. Lipids Health Dis.

[10] Hong Y, Manoharan I, Suryawanshi A (2016). Deletion of LRP5 and LRP6 in dendritic cells enhances anti-tumor immunity. Oncoimmunology.

[11] Zhu WJ, Yang SD, Qu CX (2017). Low-density lipoprotein-coupled micelles with reduction and pH dual sensitivity for intelligent co-delivery of paclitaxel and siRNA to breast tumor. Int J Nanomedicine.

[12] Han Y, Ding B, Zhao Z (2018). Immune lipoprotein nanostructures inspired relay drug delivery for amplifying antitumor efficiency. Biomaterials.

[13] Ackerman D, Simon MC (2014). Hypoxia, lipids, and cancer: surviving the harsh tumor microenvironment. Trends Cell Biol.

[14] Ma MZ, Yuan SQ, Chen YM (2018). Preoperative apolipoprotein B/apolipoprotein A1 ratio: A novel prognostic factor for gastric cancer. Oncotargets Ther.

[15] Hui C, Wei JW, Kai C (2018). Apolipoprotein A-I is a prognosticator of nasopharyngeal carcinoma in the era of intensity-modulated radiotherapy. J Cancer.

[16] Sirnio P, Vayrynen JP, Klintrup K, et al. Decreased serum apolipoprotein A1 levels are associated with poor survival and systemic inflammatory response in colorectal cancer. Sci Rep. 2017;7(1):5374. 10.1038/s41598-017-05415-9 PMC5511233 28710487

[17] McGowan-Jordan J, Simons A, Schmid M (2016). ISCN 2016: an International System for Human Cytogenomic Nomenclature (2016): S KARGER AG.

[18] Camp RL, Dolled-Filhart M, Rimm DL (2004). X-tile: a new bio-informatics tool for biomarker assessment and outcome-based cut-point optimization. Clin Cancer Res.

[19] Michalaki V, Koutroulis G, Syrigos K (2005). Evaluation of serum lipids and high-density lipoprotein subfractions (HDL2, HDL3) in postmenopausal patients with breast cancer. Mol Cell Biochem.

[20] Wilhelm AJ, Zabalawi M, Owen JS (2010). Apolipoprotein A-I modulates regulatory T cells in autoimmune LDLr−/−, ApoA-I−/− mice. J Biol Chem.

[21] Iqbal AJ, Barrett TJ, Taylor L (2016). Acute exposure to apolipoprotein A1 inhibits macrophage chemotaxis in vitro and monocyte recruitment in vivo. eLife.

[22] Zamanian-Daryoush M, Lindner D, Tallant TC (2013). The cardioprotective protein apolipoprotein A1 promotes potent anti-tumorigenic effects. J Biol Chem.

[23] Lane DM, Boatman KK, Mcconathy WJ (1995). Serum lipids and apolipoproteins in women with breast masses. Breast Cancer Res Treat.

[24] Luo X, Zhong G, Hu L (2015). Serum apolipoprotein A- I is a novel prognostic indicator for non-metastatic nasopharyngeal carcinoma. Oncotarget.

[25] Zhang L, Mcgraw KL, Sallman DA (2017). The role of p53 in myelodysplastic syndromes and acute myeloid leukemia:molecular aspects and clinical implications. Leuk Lymphoma.

[26] Cazzola M, Della PMG, Malcovati L (2013). The genetic basis of myelodysplasia and its clinical relevance. Blood..

[27] Stengel A, Kern W, Haferlach T (2017). The impact of TP53 mutations and TP53 deletions on survival varies between AML,ALL, MDS and CLL: an analysis of 3307 cases. Leukemia.

[28] Wang X, Zhao X, Gao X, Mei Y, Wu M (2013). A new role of p53 in regulating lipid metabolism. J Mol Cell Biol.

[29] Goldstein I, Rotter V (2012). Regulation of lipid metabolism by p53-fighting two villains with one swor. Trends Endocrinol Metab.

[30] Mengyi D, Min X, Deng J (2020). Evaluation of different scoring systems and gene mutations for the prognosis of myelodysplastic syndrome in Chinese population. J Cancer.

